# Disentangling multistep kinetics by combining electrochemical Arrhenius analysis with micro-kinetic modelling

**DOI:** 10.1039/d5fd00127g

**Published:** 2026-03-09

**Authors:** Mathieu Lizée, Alex Ricardo Silva Olaya, Jody Druce, Beatriz Roldan Cuenya, Sebastian Z. Oener

**Affiliations:** a Fritz Haber Institute of the Max Planck Society Berlin Germany lizee@fhi-berlin.mpg.de oener@fhi-berlin.mpg.de

## Abstract

Electrocatalytic reactions often involve several steps and intermediates, along with changes in the catalyst structure and chemistry under reaction conditions, making their mechanistic understanding very challenging. As a way to extract maximum information about the kinetics, temperature-dependent electrochemistry enables access to the apparent activation energy and pre-exponential factor as a function of the electrochemical bias. Recently, many reactions were shown to exhibit rich structures in their bias-dependent activation parameters, structures which cannot be accounted for by the traditional, single-step Butler–Volmer theory. Here, we study the overpotential-dependent activation parameters of a 2-step microkinetic model featuring an electrochemical adsorption followed by a chemical recombination step. We show that the electrochemical bias drives transitions across several kinetic regimes where the degree of rate control of each step varies. A key finding is that the bias dependent Arrhenius signatures constrain the underlying phase space of intermediate binding and activation enthalpies, even for such a seemingly simple model. From the close fit of our model with our experiments on the oxygen reduction reaction, we find that for a wide overpotential range, one kinetically relevant intermediate – and thus two partially rate determining steps – are controlling the kinetics on platinum and ruthenium nanoparticles. From the fits, we extract the corresponding binding and activation energies along with bias-dependent coverage. We argue that combining temperature dependent electrochemistry with minimalistic micro-kinetic models allows a direct comparison with DFT calculations and *operando* spectroscopy measurements.

## Abstract 2

We introduce a 2-step microkinetic model involving a single reaction intermediate and show that its bias-dependent Arrhenius signatures closely match our measurements of the oxygen reduction reaction on platinum and ruthenium nanoparticles.

## Graphical abstract

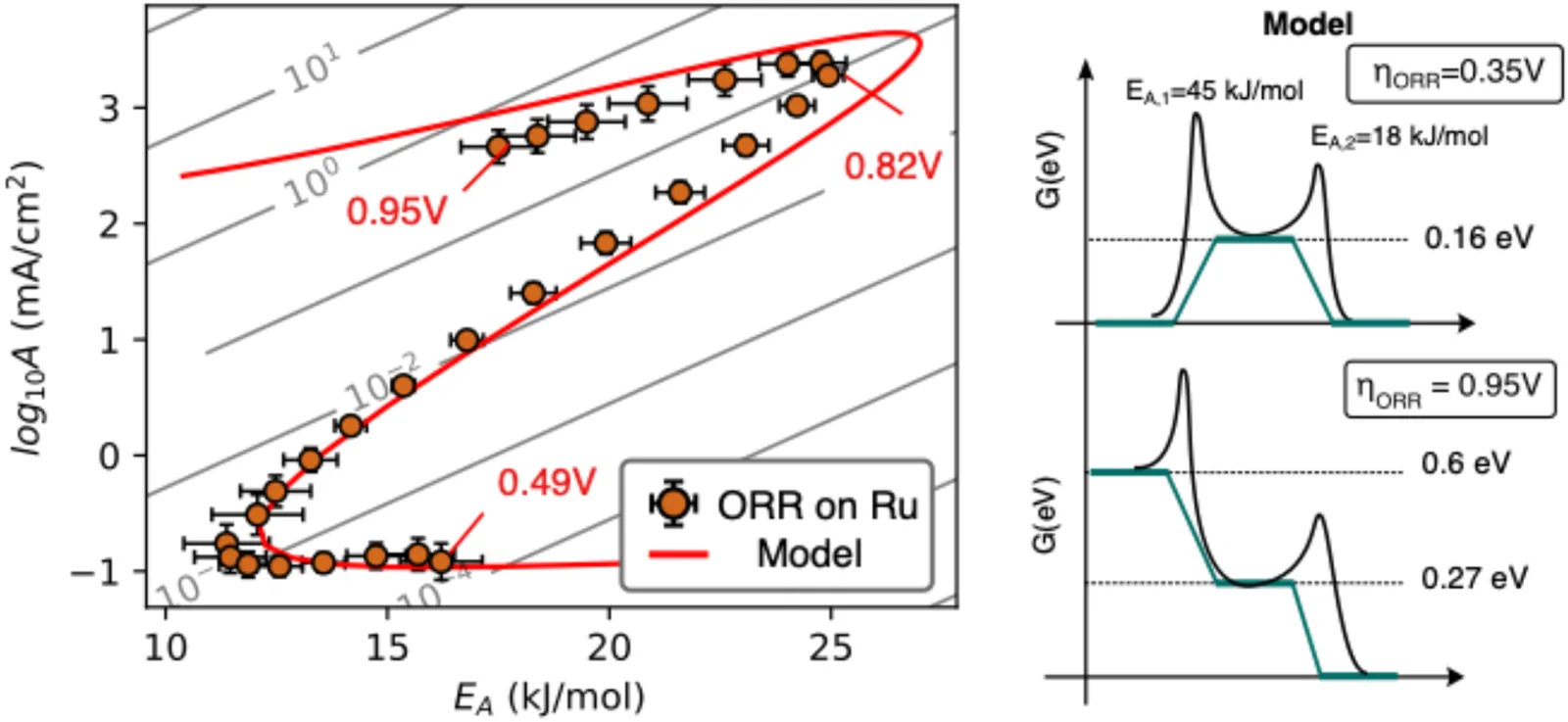

## Introduction

Economically important electrochemical reactions such as the hydrogen evolution (HER), oxygen reduction (ORR) and oxygen evolution (OER) reaction involve several steps and intermediates, making their mechanistic understanding and optimization particularly challenging. As an additional complexity with respect to thermal catalysis, the free energy driving force – the electrochemical potential difference – also affects the kinetics of single steps and the stability of intermediates at a bias-dependent catalyst-solution interphase in ways which are still poorly understood. Optimizing electrocatalysts requires a microscopic understanding of structure, chemistry and dynamics, but also a knowledge of the rate determining steps and intermediates under reaction conditions.

One powerful approach is to extract the apparent activation energy and pre-exponential factors at a given overpotential from electrochemical Arrhenius analysis. Rich behaviours have been reported experimentally. As first reported by Agar,^1^ and studied extensively by Conway,^2,3^ the activation energy and pre-exponential factor can change in unexpected ways with applied overpotential – at odds with the traditional Butler–Volmer hypothesis. Often a compensation behaviour between the apparent activation energy and the pre-exponential factor was observed, which led Conway to introduce enthalpic and entropic charge transfer coefficients, but still largely maintaining a single-step hypothesis. More recently, our group has systematically performed temperature-dependent measurements in many conditions for a wealth of reactions with various surfaces and electrolytes.^4–7^ We discovered a compensation behaviour of increasing activation energies and pre-factors at lower overpotential that appears quasi-universal across the majority of reactions and conditions. However, for multiple reactions and conditions, we observed a change in the kinetic regime, where the initial compensation region is overcome and the kinetics transition into a traditional Butler–Volmer region. On the ORR under high mass transport conditions, we observe that the bias dependent activation parameters cascade through a series of activation energy and pre-factor maxima and minima with bias that strongly suggest an overpotential-dependent degree of rate control. These changes were paralleled by bias-dependent charge transfer coefficients and Tafel slopes that can reach very high values. These results strongly suggest that the assumption of a single rate-limiting step does not hold.^7^ Therefore, the broader question arises now, how, for multi-step reactions, the bias dependent apparent activation parameters can be linked to the properties of the actual single-step transition states and intermediate binding free energies.

Micro-kinetic models have a long history in thermal catalysis^8–10^ and are especially useful to tackle mechanisms which do not have a single rate determining step but instead a few rate-determining transition states and intermediates.^9–11^ In electrochemistry, they have been used to model multistep reactions and connect the binding energy of intermediates to bias-dependent Tafel slopes.^12^ The link to Arrhenius analysis and degrees of rate control however was introduced only very recently.^13^ In this work, we model a two-step reaction with an electrochemical adsorption followed by a chemical recombination step. We compute the overpotential and temperature-dependent kinetics and find complex bias-dependent Arrhenius parameters which can be directly compared to experiments. In particular, for a wide range of parameters, the model features a strong compensation region which then turns again into a Butler–Volmer regime at higher overpotential, a behaviour which was observed experimentally, especially for the OER, ORR and HER and CO_2_RR under certain conditions.^4–7,14,15^

Here, we show that the compensation region can originate from a transition between different kinetic regimes across which the influence of the intermediate’s binding energy and other microscopic parameters vary. Our model reproduces closely our experimental results on the ORR on Pt/C and Ru/C nanoparticles, suggesting that for a wide overpotential window, two partially rate-determining transition states and one kinetically relevant intermediate might suffice to describe ORR kinetics. This work underscores the importance to consider the multistep nature of catalytic reactions to interpret electrochemical kinetics and suggests caution when using single-step transition state pictures.

## Methods

### Microkinetic model

**Fig. 1:**
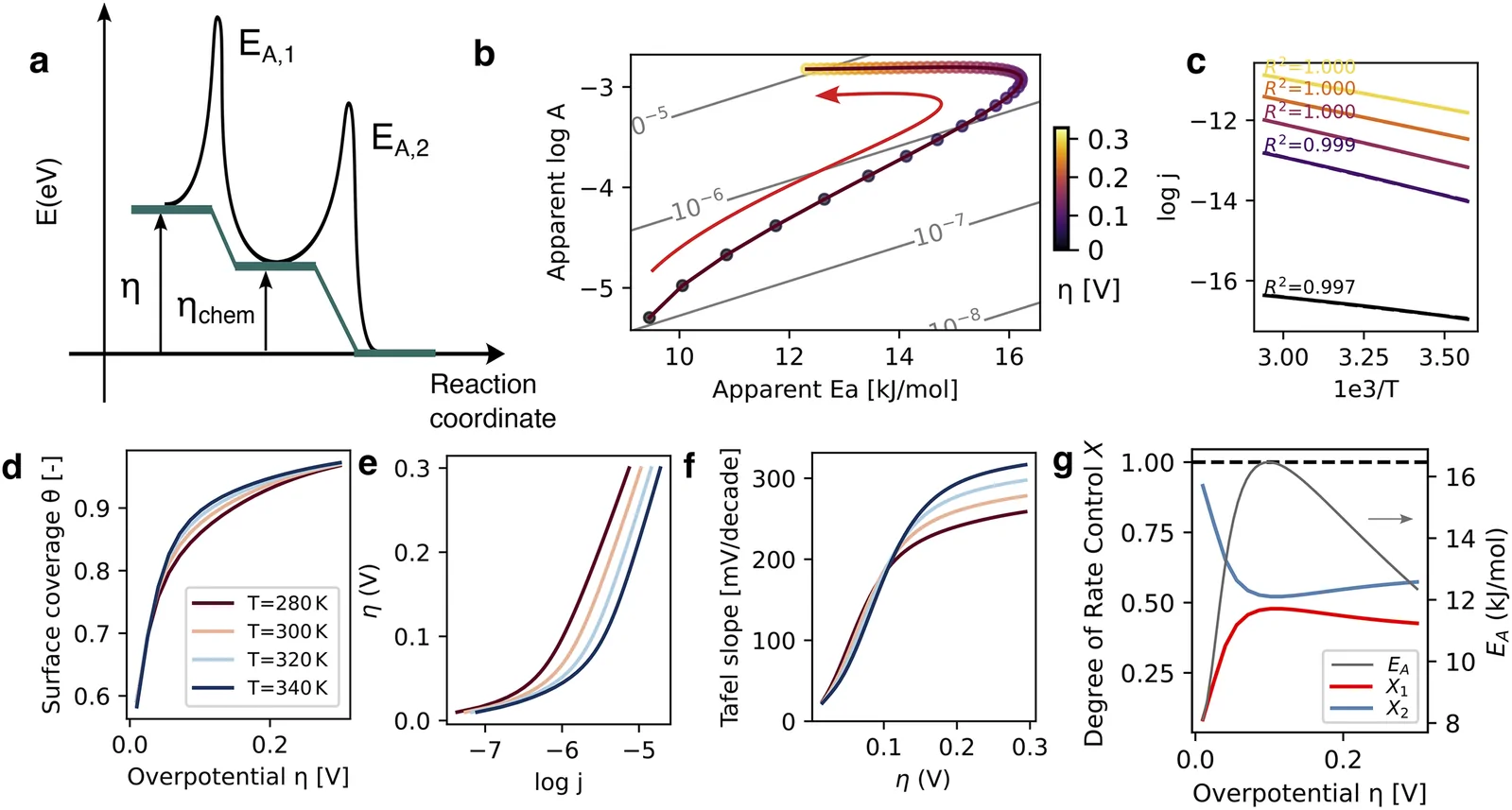
Model definition and solution for *E*_A,1_ = 30 kJ mol^−1^ and *E*_A,2_ = 10 kJ mol^−1^, ΔH* = 0, *k*_2_^0^/*k*_1_^0^ = 10^−6^. (a) Free energy diagram of the two-step microkinetic model. The free energy of adsorbed intermediates *η*_chem_, depends on the coverage *θ* and drives the recombination step. The coverage itself is determined for each overpotential and temperature by solving the steady state condition. (b) Kinetic map measured between 280 and 340 K showing the apparent pre-exponential factor log_10_*A versus* the apparent activation energy *E*_A_. The colour codes show the overpotential, which is zero in the lower left corner. log_10_*A* and *E*_A_ both increase in the compensation region. After the turnover, we find a more conventional Butler–Volmer behaviour. (c) Arrhenius plots of the current density against the inverse temperature (in K^−1^) for various overpotentials. The overpotentials are given here again by the colorscale of panel b. (d) Surface coverage *θ* as a function of the applied overpotential *η*. (e) Tafel plot of the overpotential *versus* the logarithm of the current density log_10_*j*. (f) Bias-dependent Tafel slope for *T* ∈ {280 300 320 340} K. The Tafel slope starts at relatively small values but saturates close to 300 mV per decade. (g) Degree of rate control (DRC) of steps 1 and 2 (*X*_1_and *X*_2_) as functions of the overpotential. We also plot the apparent activation energy (grey line). We plot the sum *X*_1_ + *X*_2_ as a black dashed line which is, as expected,^10^ a constant equal to 1.

Let us consider a two-step reaction mechanism with an electrochemical adsorption followed by a chemical recombination, inspired by the Volmer–Tafel mechanism of the Hydrogen Evolution Reaction (HER) (see Fig. 1a):1
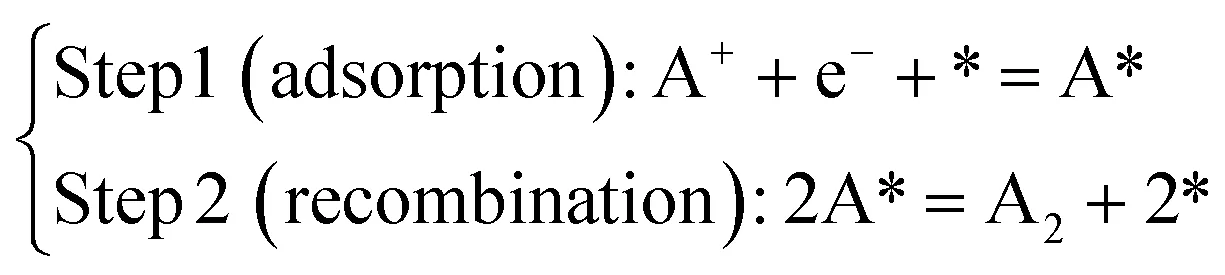
where * denotes a free site on the electrode and A* an adsorbed A atom.

We write the free energy of A* as a function of the adsorption enthalpy ΔH*, and the entropic term with a Langmuir isotherm. Following ref. 13 we define the chemical overpotential of the reaction *η*_chem_ as the free energy of A*:2
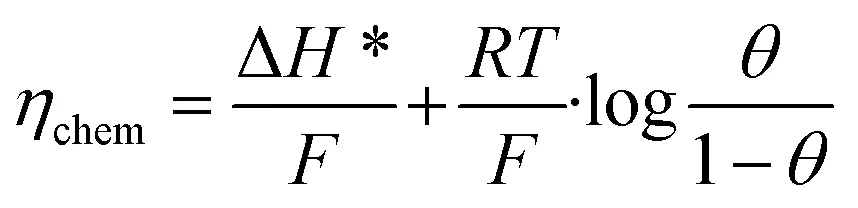
where *θ* denotes the coverage of species A* which is left as a free parameter to depend on temperature and overpotential in an *a priori* unknown way. *F* denotes the faraday constant, *R* the molar gas constant and *T* the temperature. As we detail in the subsequent section, the coverage *θ* is found by enforcing the steady state condition for the reaction.

The reaction free energy will then involve *η* − *η*_chem_ for the adsorption step and *η*_chem_ for the recombination step, hence the term ‘chemical overpotential’. Let us now assume that the activation entropy Δ*S*^+^ of both steps is constant and independent of *η* and *θ*. We can then include Δ*S*^+^ in the pre-exponential factors to obtain standard Butler–Volmer kinetics. For thermodynamic consistency, the reverse currents are fixed by the detailed balance condition (*k*_−*i*_ = *k*_*i*_·exp(−Δ*G*/*RT*)). Rates for the electrochemical adsorption step can be written:3
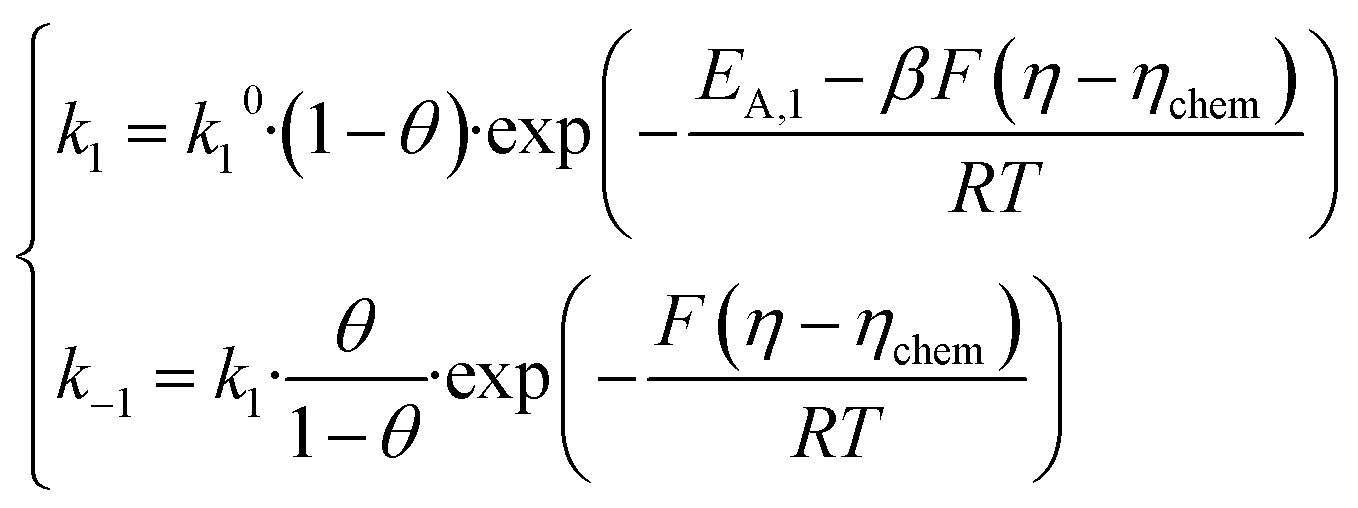
and for the chemical recombination step:4
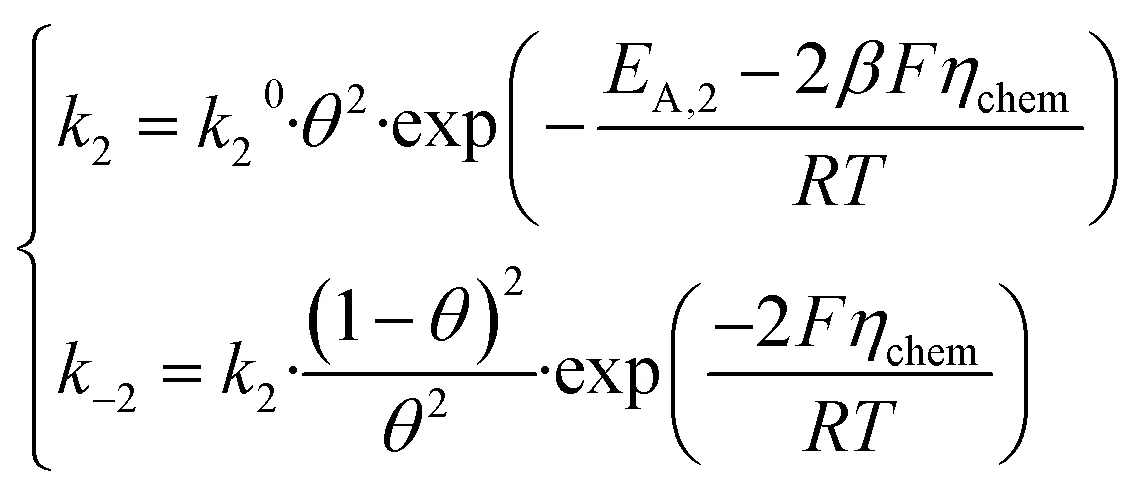


Note that the symmetry factor of barriers *β* is fixed at 0.5 throughout the paper. We intentionally keep a general expression for the single step pre-exponential factors *k*_1_^0^ and *k*_2_^0^. Although a comprehensive microscopic model will have to precisely disentangle the attempt frequency and entropic contributions to *k*_1_^0^ and *k*_2_^0^, such efforts are unnecessary in the first analysis of experimental Arrhenius data and such discussion can come later. As such, the model depends on four intrinsic parameters: the two activation energies *E*_A,1_, *E*_A,2_, the intermediate binding enthalpy ΔH*, and the ratio of pre-exponential factors *k*_2_^0^/*k*_1_^0^ which may easily be very large for steps that have very different activation entropies.^8^ In the remainder of the paper, we will fix *k*_1_^0^ = 1, and give all current densities in non-dimensional units. On top of intrinsic parameters, the solution also depends on the experimental conditions: the temperature *T* and the applied overpotential *η*.

### Solution

From this sequential, two-single step hypothesis, we use a steady-state condition, *i.e.* forcing an equal current of species A through steps 1 and 2. In practice, we fix the temperature *T* and overpotential *η*, and use a numerical solver to find the coverage *θ* that satisfies the following equation:

5 *k*_1_ − *k*_−1_ = 2·(*k*_2_ − *k*_−2_).

Solving this conservation equation gives the steady-state coverage *θ*(*η*) and current density *j*(*η*) = *k*_1_ − *k*_−1_, which is experimentally measurable. This numerical resolution typically takes about 3 ms on a standard laptop, allowing rapid exploration of the parameter space. Let us now explore the kinetic signatures of this model and compare them with experimental findings. In the following, we fix the activation energies to *E*_A,1_ = 30 kJ mol^−1^ and *E*_A,2_ = 10 kJ mol^−1^, the intermediate binding enthalpy to ΔH* = 0, and the pre-exponential factor to *k*_2_^0^ = 10^−5^ (we recall that *k*_1_^0^ is fixed at 1). We will first analyse the potential-dependent kinetics between 0 and 300 mV overpotential and temperatures between 280 and 340 K under these specific conditions. Then, we will explore in more detail the influence of the four intrinsic parameters on the potential-dependent kinetics.

## Results and discussion

By solving eqn (5) over a range of overpotentials, we obtain the surface coverage *θ*(*η*, *T*) and current density *j*(*η*, *T*), reported for *T* ∈ {280, 300, 320, 340} K in Fig. 1d and e (in the Tafel representation), respectively. As expected, both current density and coverage increase with applied overpotential *η*. Over this overpotential range, the current density increases by roughly two decades, while the coverage rises from 50% at equilibrium (entropy-controlled since ΔH* = 0) up to nearly 100% near 300 mV. Temperature has a relatively weak effect on coverage but increases the current substantially. To describe more precisely the temperature dependence as a function of overpotential, we now turn to the Arrhenius analysis.

### Arrhenius analysis

From the steady-state current density *j*(*η*, *T*) measured at several temperatures, we perform an Arrhenius analysis to extract the apparent activation energy *E*_A_(*η*) and pre-exponential factor *A*(*η*). Typical Arrhenius plots are shown in Fig. 1c for overpotentials between 0 and 300 mV (see colorbar). Interestingly, we find excellent linearity in these plots (*R*^2^ > 0.99) across the entire parameter space, despite the absence of a single well-defined rate-determining step. This observation cautions that good Arrhenius behaviour does not necessarily imply the validity of transition-state-theory assumptions. From the Arrhenius plots, we extract *E*_A_(*η*) and log_10_*A*(*η*), which we plot one *versus* the other on the kinetic map (or Constable plot) in Fig. 1b. The kinetic map is a very useful representation showing how activation energy and pre-exponential factor co-vary with applied overpotential. The diagonal grey lines are iso-current lines corresponding to perfect compensation between *E*_A_ and log_10_*A* at 300 K. On the kinetic map, we identify two distinct regimes: a compensation region at low *η* where both *E*_A_ and log_10_*A* increase strongly up to ≈150 mV and a Butler–Volmer region at high overpotential where the activation energy drops while log_10_*A* remains constant.

Very similar two-regime kinetic maps have been experimentally observed for the Oxygen Evolution Reaction (OER) at oxide catalysts^14^ and for Oxygen Reduction Reaction (ORR) on platinum.^7^ To put these results into perspective, we measure a single Butler–Volmer behaviour for HER on platinum group metals.^4^ In other instances, we measured very extended compensation regions with no turnover to the Butler–Volmer regime at high overpotential.^6^ Finally, as we later discuss in more detail, we measured more complex maps with three or more regions for the ORR on rhodium, iridium and ruthenium.^7^

### Tafel analysis

Let us now turn to the Tafel analysis, often used to connect polarization curves to reaction mechanisms and previously investigated with similar microkinetic models by Exner.^12^ We plot in Fig. 1e the overpotential as a function of the log-current density for a few temperatures *T* ∈ {280, 300, 320, 340} K. We clearly observe two regimes with relatively well-defined slopes. By differentiating these curves, we extract the Tafel slope6
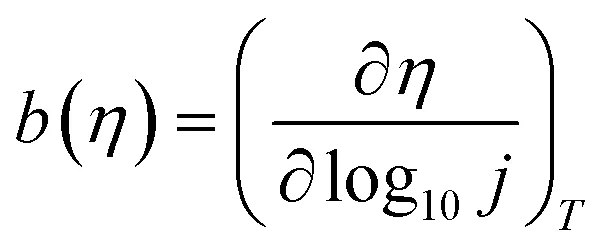
which we plot in Fig. 1f for various temperatures as a function of the overpotential *η*. We observe a non-constant Tafel slope, starting around 50 mV per dec. at low overpotential and saturating at roughly 300 mV per dec. at high overpotential. Here again, we find a well-defined Tafel slope despite the absence of a single rate-determining step. In the ‘High overpotential’ section below, we treat analytically the high overpotential limit and recover this 300 mV per dec. slope.

### Degree of rate control

Let us now analyse our model within the Degree of Rate Control (DRC) framework, introduced by Campbell for thermal catalysis reactions^9,10^ and later extended to electrochemical catalysis.^13^ The degree of rate control of a step or intermediate is defined as the derivative of the logarithm of the reaction rate with respect to the activation or binding free energy of that step or intermediate:7
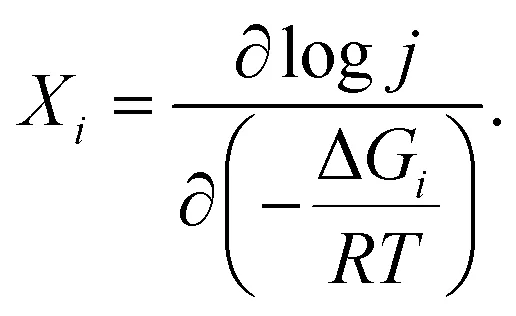
In the present model, following Campbell,^10^ we compute the degrees of rate control *X*_1_ and *X*_2_ for steps 1 and 2 by measuring the logarithmic derivative of the current density with respect to the pre-exponential factors *k*_1_^0^ and *k*_2_^0^, while keeping all other parameters constant:8
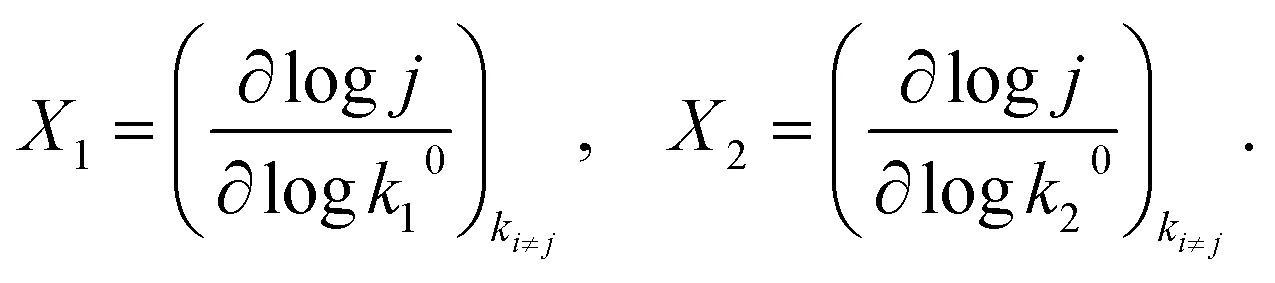


We plot *X*_1_ and *X*_2_ as functions of the overpotential *η* in Fig. 1g (red and blue lines) and the sum *X*_1_ + *X*_2_ as a black dashed line which is equal to 1, consistently with the DRC framework.^9,10,13^ At low overpotential, *X*_1_ ≈ 0 and *X*_2_ ≈ 1, indicating fully recombination-controlled kinetics. As the overpotential increases, we observe a smooth transition to mixed kinetics with *X*_1_ ≈ 0.4 and *X*_2_ ≈ 0.6 above roughly 100 mV. We also plot the apparent activation energy *E*_A_(*η*) (grey line). It appears that the high overpotential Butler–Volmer regime—corresponding to the decreasing *E*_A_ region above 150 mV—matches the plateau in the DRC curves. This observation is significant because, despite (1) excellent Arrhenius fits, (2) a well-defined Tafel slope, and (3) clear Butler–Volmer behaviour, the high overpotential kinetics are not controlled by a single step, making it difficult to extract meaningful mechanistic parameters (like activation enthalpy or entropy of a “transition state”). This cautions against applying transition state theory—which assumes a single, well-defined transition state—unless this assumption is very well justified. This correlation between the kinetic map and degrees of rate control already positions Arrhenius analysis as a very powerful tool to disentangle multistep electrochemical mechanisms. We will show below that the Arrhenius apparent pre-exponential factor is almost a perfect proxy for *X*_1_.

### High overpotential limit

We will now rationalize the high overpotential trends observed until now: a Tafel slope of 300 mV per dec. and a degree of rate control of step 2 *X*_2_, equal to 0.6. At high overpotential, the reverse rates become negligible. From the steady state condition eqn (5), we find *η*_chem_ at high applied overpotential:9
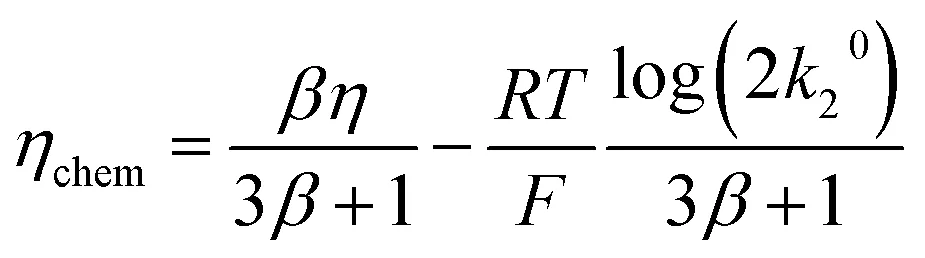
and write the current density:10
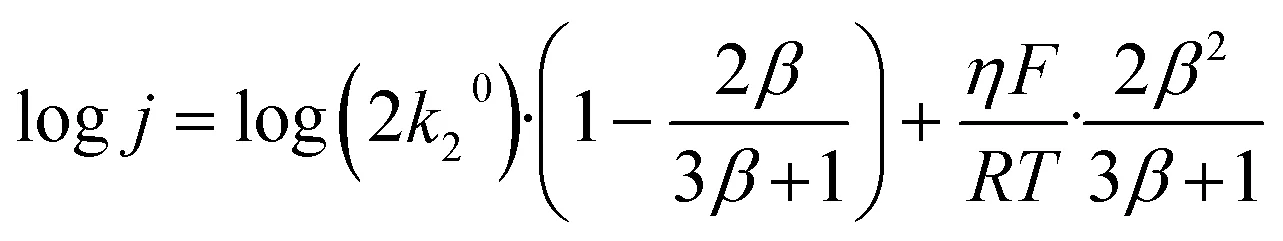
and find the Tafel slope11
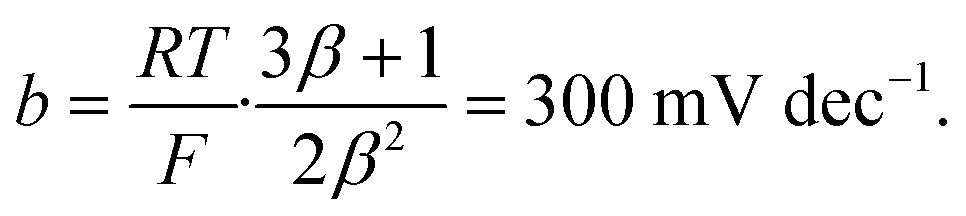


The degree of rate control of step 2 at high overpotential becomes12
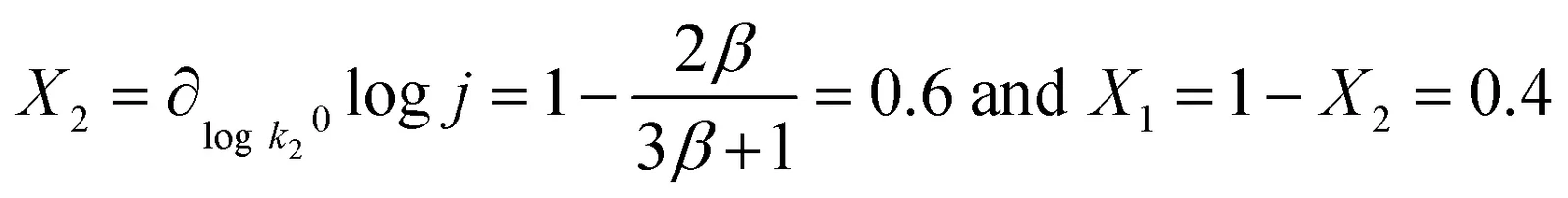
as observed numerically. Eqn (10) also leads to a scaling of pre-exponential factor and coverage with *k*_2_^0^,13
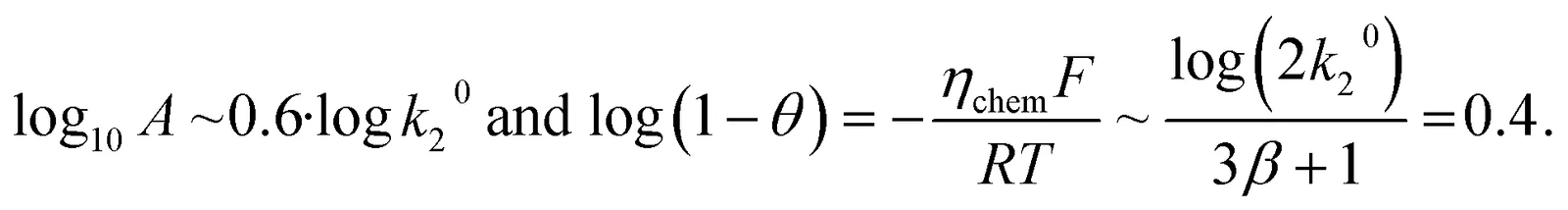


**Fig. 2:**
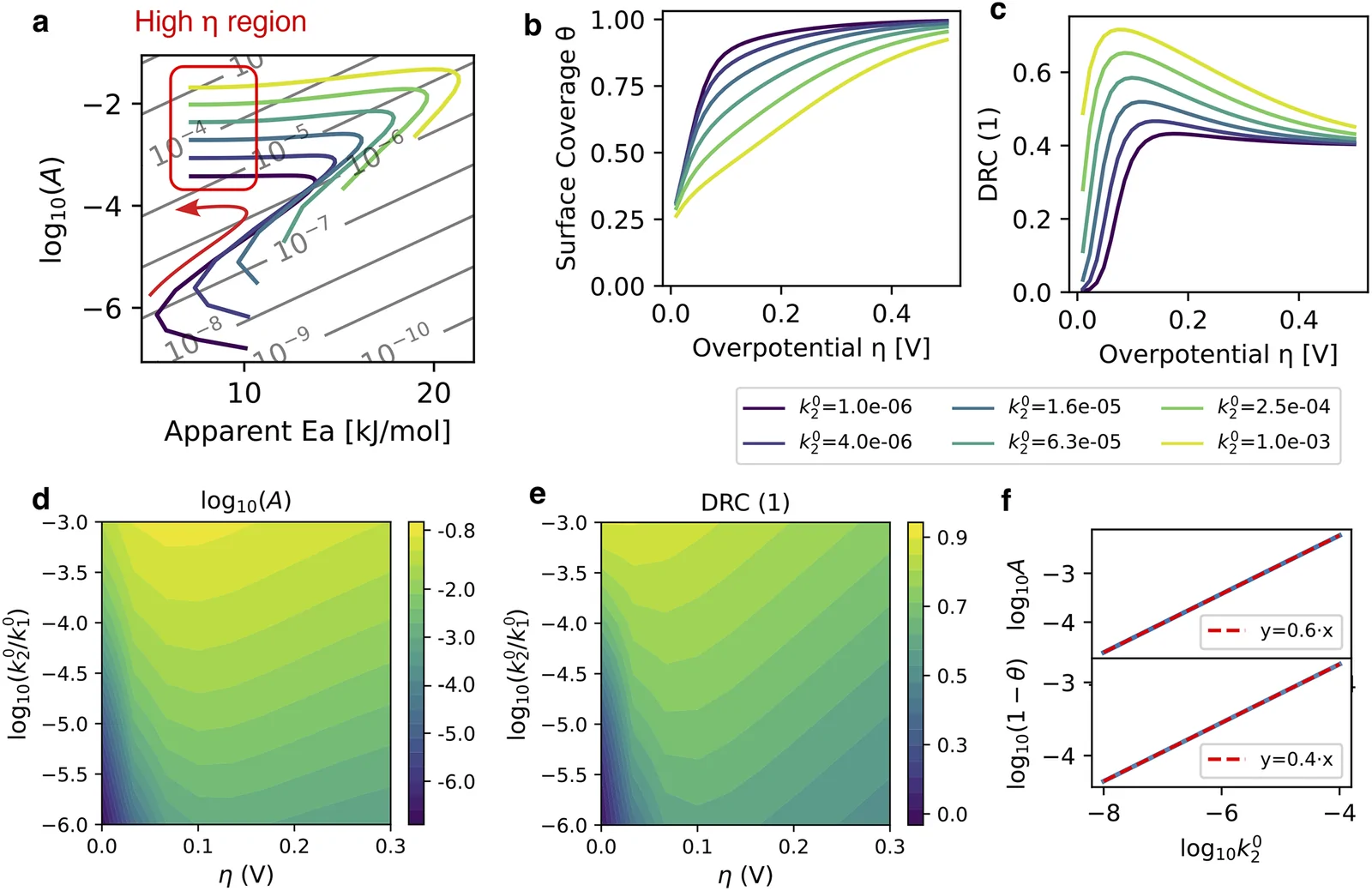
Influence of the relative pre-exponential factors *k*_2_/*k*_1_^0^ on the kinetics for an intermediate binding enthalpy ΔH* = 30 meV, with activation enthalpies fixed at *E*_A,1_ = 30 kJ mol^−1^ and *E*_A,2_ = 10 kJ mol^−1^. The overpotential is swept continuously between 0 and 300 mV, and *k*_2_^0^ is varied between 10^−6^ and 10^−3^. (a) Kinetic maps: pre-exponential factor log_10_*A versus* apparent activation energy *E*_A_. Colour code for *k*_2_^0^ and the red box highlights the high overpotential limit where degrees of rate control, Tafel slope, log_10_*A* and coverage are known analytically. (b) Surface coverage *θ* as a function of overpotential for *k*_2_^0^ (as in the colour key below). (c) Degree of rate control of the adsorption step 1 *X*_1_. (d) Colormap of the apparent pre-exponential factor log_10_*A*. as a function of overpotential and of log_10_*k*_2_^0^. Horizontal cuts are overpotential-dependent curves for a given set of microscopic parameters. (e) Degree of rate control of the first step *X*_1_. Note the very strong resemblance to the pre-exponential factor map on panel d, showing that log_10_*A* is an excellent proxy for *X*_1_. The dark region at low overpotential is the recombination-controlled regime which is especially strong for very low *k*_2_^0^. In the high overpotential limit, on the right side of the map, *X*_1_ tends towards 0.4 (see eqn (12)). (f) Numerical test of the high *η* scaling relations eqn (3) for the pre-exponential factor log_10_*A* and the coverage log_10_(1 − *θ*) against log_10_*k*_2_^0^. Numerical results (blue solid line) are obtained at *η* = 1 V and the linear fits (red solid lines) have slopes that perfectly match eqn (13).

Both these power laws are verified numerically with high accuracy in Fig. 2f where we plot log_10_*A* and log_10_(1 − *θ*) *versus* log_10_*k*_2_^0^ at a high overpotential (*η* = 1 V). The non-vanishing rate control of the Volmer step at high overpotential may seem counter-intuitive: it is actually a direct consequence of the Langmuir isotherm through which the Volmer rate enhances the intermediate's activation energy and thus the recombination rate even at high coverage (*θ* ∼ 1). If one were to assume a coverage-independent intermediate free energy (*η*_chem_ = cste), the DRC of the Volmer step would vanish and the Tafel slope would diverge.

### Influence of the relative pre-exponential factors

To further explore the influence of the model’s intrinsic parameters on kinetics, we first vary the recombination step’s pre-exponential factors *k*_2_^0^ between 10^−5^ and 10^−2^ (keep in mind that *k*_1_^0^ = 1), keeping activation enthalpies fixed at *E*_A,1_ = 30 kJ mol^−1^ and *E*_A,2_ = 10 kJ mol^−1^ and the intermediate binding enthalpy to ΔH* = 30 meV. We show in Fig. 2 the potential-dependent kinetics for *k*_2_^0^/*k*_1_^0^ ∈ {10^−5^, 10^−4^, 10^−3^, 10^−2^} with the kinetic maps, the surface coverage and the DRC of step 1 in panels a–c. Overall, we see that increasing the relative rate of the recombination step lowers the coverage and the Tafel slope while shifting kinetic maps to higher apparent activation energies and pre-exponential factors. At the turnover potential, we find that the DRC of the adsorption step is much higher for high *k*_2_^0^/*k*_1_^0^ ratios, explaining the high activation energy, closer to *E*_A,1_ than *E*_A,2_.

We also plot the pre-exponential factors and the degree of rate control of step 1 as 2-dimensional colormaps on panels d–e. From the perfect correlation of log_10_*A* and *X*_1_ (see panels c–d), we conclude that the compensation phenomenology is linked to the strong enhancement of the pre-exponential factor along the transition from the recombination-controlled regime. This 1-to-1 correspondence between the pre-exponential factor and the DRC of step 1 *X*_1_ is a very useful rule of thumb to keep in mind from the model analysis and which directly applies to the analysis of HER kinetics on coinage metals where the microscopic hypothesis of the model are quite accurate and where we reported strong compensation.^4^ In this framework, the pre-exponential factor is a direct measure of the degree of rate control of step 1.

### Overpotential-dependent volcano plot

**Fig. 3:**
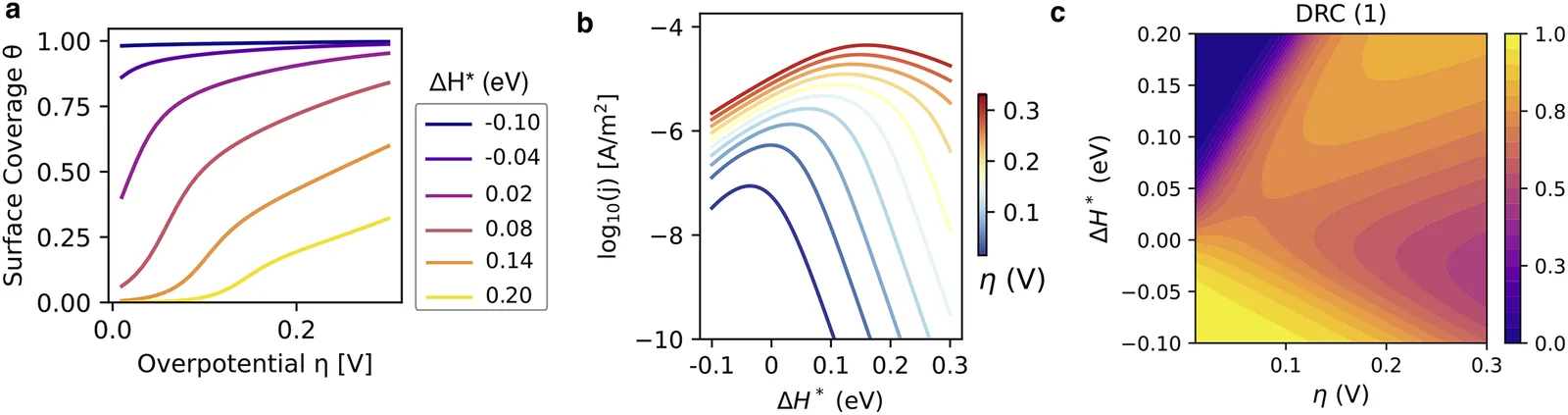
Influence of the intermediate binding enthalpy ΔH* on the kinetics for a ratio of pre-exponential factors *k*_2_^0^/*k*_1_^0^ = 10^−5^, with activation enthalpies fixed at *E*_A,1_ = 30 kJ mol^−1^ and *E*_A,2_ = 10 kJ mol^−1^. The overpotential is swept continuously between 0 and 300 mV, and the intermediate binding enthalpy is varied between −0.1 and 0.3 eV. (a) Surface coverage *θ*. (b) Volcano plot: current density *versus* intermediate binding energy for various overpotentials. (c) Heat map of the degree of rate control of the first step *X*_1_ as a function of overpotential (*x* axis) and intermediate binding enthalpy (*y*-axis). Horizontal cuts are overpotential-dependent DRC curves *X*_1_(*η*) as shown on Fig. 1g and 2c. Vertical cuts show the influence of intermediate binding energy at a fixed overpotential.

In Fig. 3, we show the impact of the intermediate binding enthalpy ΔH* on bias-dependent kinetics. We find that the intermediate binding enthalpy ΔH* has a very strong influence on all kinetic signatures, and first and foremost on the coverage which is strongly enhanced at negative enthalpy (see panel a). To assess the role of ΔH* on kinetics as a function of bias, we plot the current density as a function of ΔH* for various overpotentials (see colorbar) on Fig. 3b. Each curve corresponds to a fixed-overpotential volcano-plot where one intermediate binding enthalpy maximizes the current at a given overpotential. The volcano shows a potential-dependent optimum, close to 0 at equilibrium and up to 0.2 eV at *η* = 300 mV. We emphasize the importance of considering a volcano plot at the relevant overpotential: here the current density at 300 mV for the equilibrium optimum (top of blue curve) is lower by one decade compared to the 300 mV optimum (top of red curve). On panel c, we turn to the degree of rate control *X*_1_ plotted as a 2d colormap. It is clear that the intermediate binding enthalpy ΔH* has a very strong influence on kinetics and can widely modulate the low overpotential kinetics by tuning the relative degrees of rate control of the adsorption and recombination steps. At very high binding enthalpy, we clearly see a fully recombination-controlled region at low overpotential (black region in upper-left corner) turning into highly adsorption-controlled kinetics close to 150 mV. The transition region in between corresponds to the compensation region. In contrast, for negative ΔH* (surface-binding intermediates), *X*_1_ remains close to its high overpotential value (0.4) and no kinetic transition is observed.

### Influence of *E*_A,1_

**Fig. 4:**
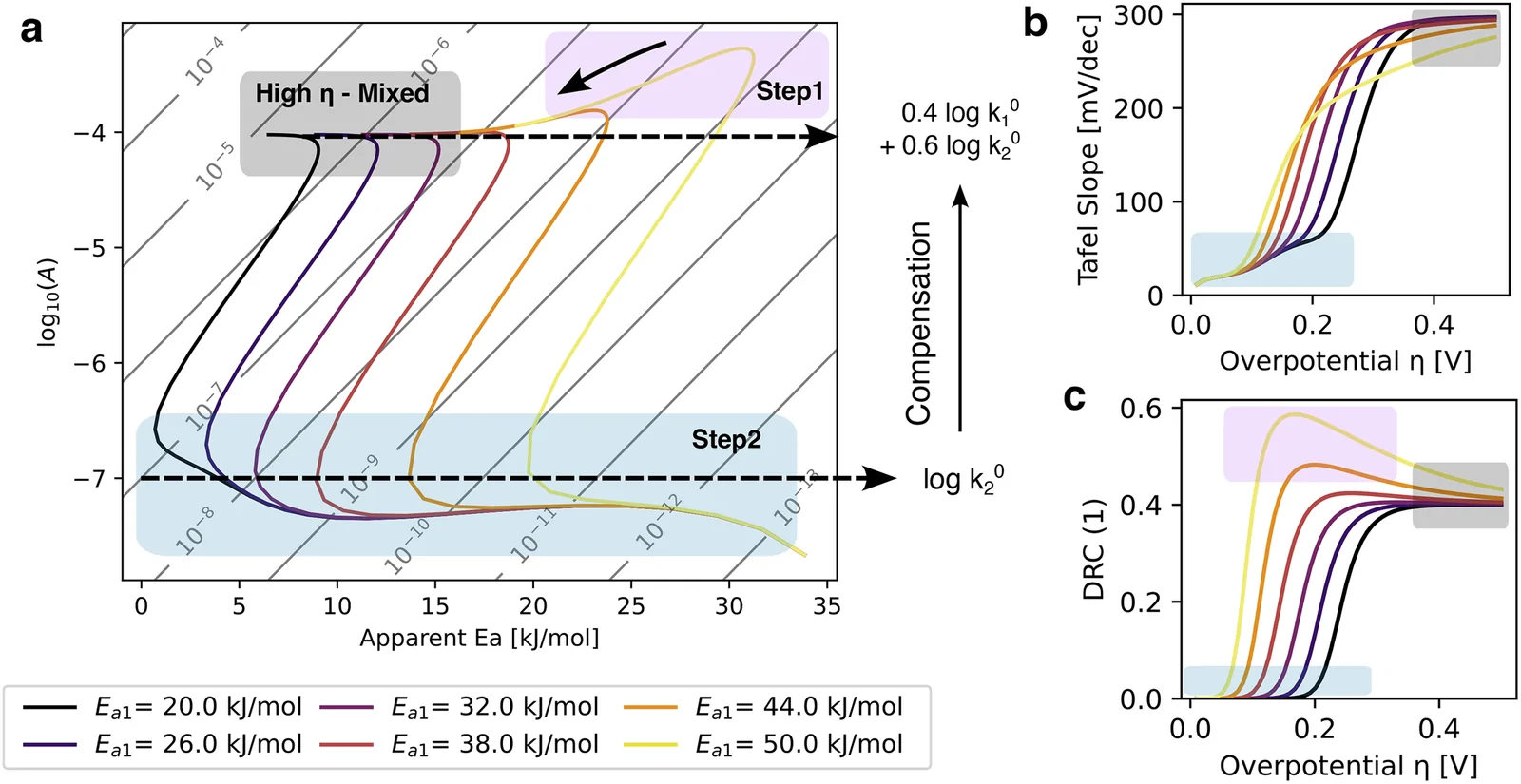
Influence of *E*_A,1_ on the overpotential-dependent kinetics with an intermediate binding enthalpy fixed at ΔH* = 100 meV, *k*_2_^0^ = 10^−7^, and activation enthalpy *E*_A,2_ = 20 kJ mol^−1^. The overpotential is swept continuously between 0 and 300 mV, and the activation enthalpy of step 1 (*E*_A,1_) is varied between 10 and 50 kJ mol^−1^ (see legend). (a) Kinetic maps: pre-exponential factor log_10_*A versus* apparent activation energy *E*_A_. The blue shaded area shows the region where the degree of rate control of the recombination step *X*_2_ (which is equal to 1 − *X*_1_) is close to 1, the pink region where *X*_1_ is higher than its high overpotential limit (0.4) and finally the grey shaded area denotes the high overpotential limit. The curves for various activation energies are found to fall on the same master curve in the low and high overpotential regimes. (b) Tafel slope *versus* overpotential for various *E*_A,1_, the colours of the curves and shaded regions are the same as on panel a. Note the high overpotential limit where the Tafel slope reaches 300 mV per dec. following eqn (11). (c) Degree of rate control of the first step *X*_1_ as a function of overpotential for various activation energies. Here again, colours are the same as panel a. We confirm the recombination controlled-regime at low overpotential and the mixed high overpotential regime where *X*_1_ → 0.4 following eqn (12).

Turning to the influence of single-step activation energies, we vary *E*_A,1_ between 20 and 50 kJ mol^−1^ while keeping a fixed *E*_A,2_ = 20 kJ mol^−1^ and *k*_2_^0^/*k*_1_^0^ = 10^−7^ on Fig. 4. Strikingly, we find a universal behaviour at high and low overpotential where all kinetic maps collapse onto a single master curve (panel a). At low overpotential, the recombination step fully dominates kinetics (*X*_1_ = 0, blue shaded area). At high overpotential, we recover the previously derived universal behaviour with *X*_1_ = 0.4 (eqn (12), grey shaded area). The transition between these two regimes occurs at lower overpotential for higher *E*_A,1_ (panel c) and shows a high *X*_1_ region (pink shaded area) for high *E*_A,1_ (here up to 0.6 but it can reach 1) where the adsorption step becomes more prominent and which has a characteristic downward trend in the kinetic map (see black arrow). Note that the downwards trend has been reported experimentally, as we shall discuss below with ORR electrochemical Arrhenius measurements. The Tafel slope (panel b) is correspondingly strongly influenced by *E*_A,1_ in the transition region and can show a plateau at 120 mV per dec. if *X*_1_ = 1 but converges to the same high overpotential limit of 300 mV per dec. From the universal behaviour described on Fig. 4a and the findings that the apparent pre-exponential factor goes log_10_*k*_2_^0^ at low overpotential to 0.6 log_10_*k*_2_^0^ + 0.4 log_10_*k*_1_^0^ at high overpotential (eqn (13)), we find that the extent of the compensation region along the *y*-axis is directly given by 0.4 log_10_*k*_1_^0^/*k*_2_^0^. As such, the key condition to observe compensation is to have a large difference in the log of pre-exponential factors *k*_2_^0^ ≪ *k*_1_^0^.

### Fitting experimental data

Let us now compare our model with experimental measurements of the ORR. We performed ORR measurements in acidic medium at well controlled oxygen pressure and high mass-transport conditions using a Nafion-based membrane-electrode assembly with platinum and ruthenium nanoparticles deposited on carbon as a catalyst, as detailed elsewhere.^7^ Although we have no clear *a priori* reason to expect that the model’s hypothesis will fully apply to the complex multi-intermediate ORR mechanism,^16^ we find an excellent agreement with the experimental data, with the same H-binding energy for both metals and realistic values for activation energies. Note that we fit the experimental data in a completely agnostic way concerning the nature of the intermediate, we view the microkinetic model as a way to drastically reduce the dimensionality of the dataset, by converting electrochemical Arrhenius measurements into a handful of intrinsic parameters which can then be compared to results from DFT calculations.^17^

**Fig. 5:**
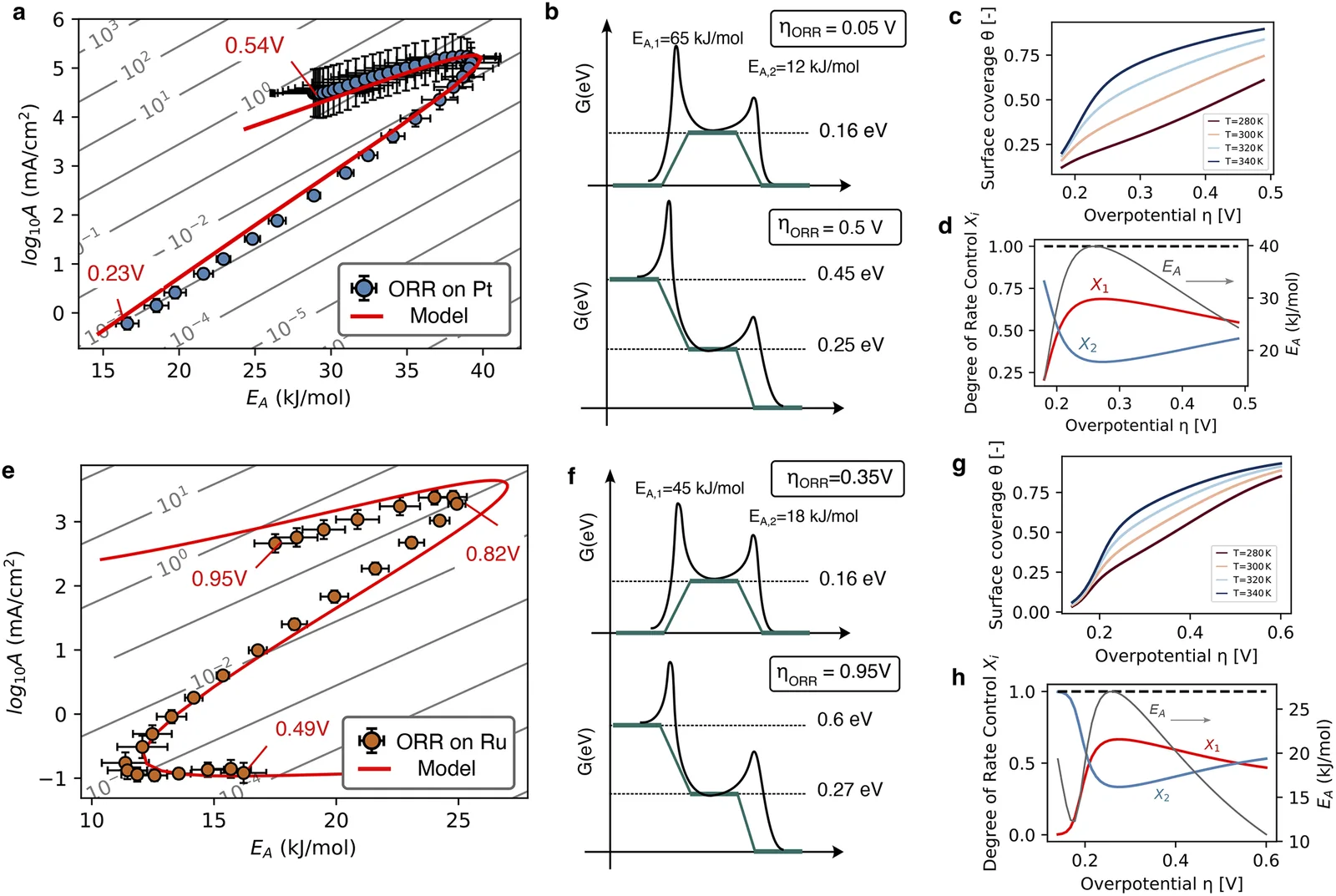
Comparison with experimental measurements of the Oxygen Reduction Reaction (ORR) on metallic nanoparticles. Measurements have been performed in a membrane-electrode assembly with a 2 bars partial pressure of O_2_. Details of the experimental protocol are given in ref. 7. (a) Kinetic map for platinum nanoparticles, the pre-exponential factor is given in units of an apparent current density mA cm^−2^ in terms of the geometrical membrane area. The red line is a solution of the present model for *E*_A,1_ = 65 kJ mol^−1^, *E*_A,2_ = 12 kJ mol^−1^, ΔH* = 0.22 eV and *k*_2_^0^/*k*_1_^0^ = 1 × 10^−12^. The overpotential *η* is shifted by 50 mV with respect to the ORR overpotential *η*_ORR_ (red labels). (b) Schematic of the free energy diagram and activation enthalpies at ORR overpotentials of 50 and 500 mV respectively. (c) Surface coverage as a function of overpotential for several temperatures. (d) Degrees of rate control *X*_1_ and *X*_2_ corresponding to the solid red-line of panel b. The apparent activation energy of the model is plotted as a grey solid line. (e) Kinetic map for ruthenium nanoparticles. The red line is a solution of the present model for *E*_A,1_ = 45 kJ mol^−1^, *E*_A,2_ = 18 kJ mol^−1^, ΔH* = 0.22 eV and *k*_2_^0^/*k*_1_^0^ = 2 × 10^−8^. The overpotential *η* shifted by 0.35 V with respect to the ORR overpotential *η*_ORR_ (red labels). (f) Schematic of the free energy diagram and activation enthalpies at ORR overpotentials of 350 and 950 mV, respectively. (g) Surface coverage as a function of overpotential for several temperatures. (h) Degrees of rate control *X*_1_ and *X*_2_ corresponding to the solid red-line of panel e. The apparent activation energy of the model is plotted as a grey solid line.

We plot the experimental kinetic map for Pt nanoparticles in Fig. 5a, with the model’s fit as a red solid line (*E*_A,1_ = 65 kJ mol^−1^, *E*_A,2_ = 12 kJ mol^−1^, ΔH* = 0.22 eV and *k*_2_^0^/*k*_1_^0^ = 1 × 10^−12^, *η* = *η*_ORR_ − 50 mV). The energy shift between the overpotential of the ORR *η*_ORR_ and that of the 2-step model *η* is due to the steps and intermediates of the real ORR mechanism which do not appear explicitely in the 2-step model. The free energy diagram corresponding to this model is shown in panel *b* for ORR overpotentials of 50 mV and of 500 mV. Note that we assume that the ORR overpotential is related to that of the two-step model by a shift of 50 mV. The intermediate free energies in these two cases are respectively 0.16 and 0.25 eV. This increase in activation energy naturally comes from the Langmuir isotherm entropic contribution at increasing coverage (eqn (2)). We show the bias-dependent coverage for several temperatures on panel c which is very strongly increasing from small (non-binding intermediate since ΔH* > 0) to almost full coverage at high overpotential. Note also that this model accounts for the downward trend on the second Butler–Volmer region, which we relate in panel d, to a relatively high degree of rate control for the electrochemical adsorption step *X*_1_ which drops towards 0.4 at with increasing bias in line with the direct correspondence between the apparent pre-exponential factor log_10_*A* and *X*_1_ that we reported in Fig. 2d–e.

Regarding ruthenium measurements (panel e), the qualitative shape of the kinetic map is very different to platinum: there is a clear Butler–Volmer region at low overpotentials, a first turnover to the compensation region and a second one to a high *X*_1_ region and then to the high-bias regime. As shown with the red line, we can fit the model to this behaviour (*E*_A,1_ = 45 kJ mol^−1^, *E*_A,2_ = 18 kJ mol^−1^, ΔH* = 0.22 eV and *k*_2_^0^/*k*_1_^0^ = 2 × 10^−8^, *η* = *η*_ORR_ − 0.35 V). Here we assume that the model’s overpotential is shifted by as much as 350 mV with respect to the ORR overpotential. From this experimental-model agreement, we conclude that the ORR kinetics in this potential range are impacted by a single kinetically-relevant intermediate and two partially rate controlling steps. Note that we assumed a fully chemical recombination step 2. We also solved the model for an electrochemical recombination (a Heyrovsky-like step) and generally found similar trends, with lower Tafel slopes at high bias.

## Conclusions

In this work, we have shown that some of the overpotential-dependent Arrhenius signatures reported experimentally over several systems can be reproduced by a 2-step electrochemical mechanism with a single kinetically-relevant intermediate species. We have shown that the various regimes identified on the ‘kinetic map’ can be mapped to kinetic regimes with various degrees of rate control for each of the two steps. Consistent with the simple assumptions of our model, we report volcano plots for the current density *versus* the intermediate binding energy with maxima that strongly shift towards less binding surfaces at higher overpotential. Finally, we used the model to fit our experimental results on the oxygen reduction reaction at platinum and ruthenium nanoparticles and found that over a wide overpotential window, the kinetic data can be rationalized in terms of two steps and a single kinetically-relevant intermediate. The combination of bias-dependent Arrhenius analysis with light and transparent microkinetic modelling promises a fruitful interaction with *operando* spectroscopy and microscopy to validate or refine kinetic hypotheses in close feedback loops and narrow down the enormous phase-space of binding energies and coverages, transition state activation enthalpies and entropies, allowing a direct comparison with quantum chemistry calculations.

## Author contributions

ML conceived of the project and performed all simulations. ARSO and JD performed all experiments and analyzed the experimental data. ML and SZO analyzed the results of the model and discussed them with BRC. ML wrote the manuscript with help from SZO and BRC.

## Conflicts of interest

There are no conflicts to declare.

## Data Availability

The data presented in this paper can be reproduced with an elementary code described in the paper.
